# Das One-Health-Konzept im Kontext globaler Warenketten, Krisen und der Sicherheit von Lebens- und Futtermitteln

**DOI:** 10.1007/s00103-023-03714-3

**Published:** 2023-05-31

**Authors:** Anneluise Mader, Oliver Riede, Ulrike Pabel, Jessica Dietrich, Katharina Sommerkorn, Robert Pieper

**Affiliations:** Abteilung Sicherheit in der Nahrungskette, Bundesinstitut für Risikobewertung (BfR), Max-Dohrn-Str. 8–10, 10589 Berlin, Deutschland

**Keywords:** Chemische Gefahren, Biologische Gefahren, Pflanzen- und Mykotoxine, Persistente organische Kontaminanten, Toxische Elemente, Chemical hazards, Biological hazards, Plant and mycotoxins, Persistent organic pollutants, Toxic elements

## Abstract

Die ganzheitliche Betrachtung der Lebens- und Futtermittelsicherheit unter Einbeziehung der Tiergesundheit und der Umweltbedingungen ist eine wichtige Säule des One-Health-Ansatzes. Die Begrifflichkeit geht damit deutlich über die häufig darunter verstandene Vermeidung der Verbreitung von übertragbaren mikrobiologischen Krankheiten hinaus und verdeutlicht, dass Mensch, Tiere und Umwelt sowie ihre Interaktionen in einem transdisziplinären Kontext betrachtet werden sollten.

In dem vorliegenden Diskussionsbeitrag zum One-Health-Ansatz liegt der Fokus weniger auf mikrobiologischen Risiken, sondern es wird insbesondere der Zusammenhang zu chemischen Risiken in der Nahrungskette hergestellt. Dieser wird an konkreten Beispielen chemischer Kontaminanten (Metalle, persistente organische Kontaminanten und natürliche Toxine) verdeutlicht und die Mechanismen von Eintrag und Weitergabe entlang der Nahrungsketten werden vorgestellt.

Die Minimierung des Vorkommens chemischer Kontaminanten und somit der Exposition erfordert internationale und interdisziplinäre Zusammenarbeit. Klimawandel, Pandemien, Rohstoffknappheit, Energiemangel, politische Krisen und Umweltkatastrophen können die gesamte Kette der Primärproduktion pflanzlicher und tierischer Lebensmittel sowie die Weiterverarbeitung und Bereitstellung der Produkte für Verbraucherinnen und Verbraucher beeinträchtigen. Neben der sich verändernden Verfügbarkeit kann dies auch einen Einfluss auf die Zusammensetzung, Qualität und Sicherheit der Lebens- und Futtermittel haben. Anhand der Effekte auf globalen Warenketten werden vulnerable und resiliente Bereiche sichtbar. Im Sinne des One-Health-Ansatzes gilt es, die Sicherheit und Resilienz entlang der Nahrungskette zu erhöhen und deren Vulnerabilität zu minimieren.

## Abstract (en)

The holistic view of food and feed safety, including animal health and environmental conditions, is an important pillar of the One Health approach. The terminology thus clearly goes beyond the prevention of spreading microbiological diseases, in which context it is often understood, and highlights that humans, animals, and the environment as well as their interaction should be considered in a transdisciplinary context.

In terms of One Health, this discussion paper focuses less on microbiological risks, but rather on the connection to chemical risks in the food chain. This is illustrated by concrete examples of chemical contaminants (metals, persistent organic contaminants, natural toxins). The mechanisms of input and transfer along the food chain are presented.

Minimizing the presence of contaminants and thus exposure requires international and interdisciplinary cooperation in the spirit of the One Health approach. Climate change, pandemics, shortages of raw materials, energy deficiencies, political crises, and environmental disasters can affect the entire food chain from primary production of plant and animal foods to further processing and provision of products to consumers. In addition to changing availability, this can also have an impact on the composition, quality, and safety of food and feed. Based on the effect on global commodity chains, vulnerable and resilient areas along the food chain become visible. In terms of the One Health approach, the aim is to increase safety and resilience along the food chain and to minimize its vulnerability.

## Einleitung

Gemäß der Definition des One Health High-Level Expert Panel (OHHLEP) der Ernährungs- und Landwirtschaftsorganisation der Vereinten Nationen (FAO), der Weltorganisation für Tiergesundheit (OIE), des Umweltprogramms der Vereinten Nationen (UNEP) und Weltgesundheitsorganisation (WHO) ist „One Health“ zu verstehen als: „*…* ein kollektiver, vereinender Ansatz, der darauf abzielt, die Gesundheit von Menschen, Tieren und Ökosystemen nachhaltig ins Gleichgewicht zu bringen und zu optimieren. Er erkennt an, dass die Gesundheit von Menschen, Haus- und Wildtieren, Pflanzen und der weiteren Umwelt (einschließlich der Ökosysteme) eng miteinander verbunden und voneinander abhängig sind. Der Ansatz mobilisiert verschiedene Sektoren, Disziplinen und Gemeinschaften auf unterschiedlichen Ebenen der Gesellschaft, um gemeinsam das Wohlergehen zu fördern und Bedrohungen der Gesundheit und der Ökosysteme zu bekämpfen und gleichzeitig den kollektiven Bedarf an sauberem Wasser, Energie und Luft sowie an sicheren und nahrhaften Lebensmitteln zu decken, Maßnahmen gegen den Klimawandel zu ergreifen und zu einer nachhaltigen Entwicklung beizutragen“ [1].

Die Begrifflichkeit geht damit deutlich über die häufig darunter verstandene Vermeidung der Verbreitung von übertragbaren mikrobiologischen Krankheiten hinaus und verdeutlicht, dass Mensch, Tiere und Umwelt sowie ihre Interaktionen in einem transdisziplinären Kontext betrachtet werden sollten, in dem Biodiversität und Zugang zu Platz und Ressourcen gleichermaßen gesichert sind. Die Notwendigkeit, diese Zusammenhänge zukünftig stärker zu berücksichtigen, wurde in den letzten Jahren besonders deutlich, beispielsweise durch die SARS-CoV-2-Pandemie, die Situation in der Ukraine (und die damit verbundenen Verschiebungen globaler Lieferketten, Verknappung von Agrarrohstoffen und Verfügbarkeit von Energie), aber auch durch den Klimawandel und die damit verbundenen globalen oder auch regionalen Ereignisse (Flutereignisse, Hitze und Dürre, Algenblüten).

Sowohl bei der Primärproduktion als auch bei der Weiterverarbeitung können Kontaminationen mit lebensmittelassoziierten (Zoo‑)Pathogenen auftreten. Eine Persistenz von der Primärproduktion entlang der Warenkette bis hin zum tierischen Lebensmittel ist für pathogene *Escherichia-coli-*Stämme, *Salmonella* ssp. und *Campylobacter* ssp. bekannt [2]. Im Folgenden soll der One-Health-Ansatz jedoch weniger im bislang häufig verstandenen Hinblick auf die Verbreitung von Krankheiten durch (Zoo‑)Pathogene diskutiert werden, vielmehr soll an konkreten Beispielen verdeutlicht werden, dass chemische Kontaminanten natürlichen oder anthropogenen Ursprungs ebenfalls unter diesen Aspekt fallen. Auch globale Warenketten und Krisen haben einen Einfluss auf deren Eintrag, Verteilung und Anreicherung in Ökosystemen, was wiederum direkte Auswirkungen auf die globale Futtermittel- und Lebensmittelsicherheit hat.

## Kontaminanten in der Nahrungskette im Sinne des One-Health-Konzepts

### Chemische Risiken in der Nahrungskette

Die Erzeugung sicherer Lebensmittel und sicherer Futtermittel für Nutztiere unterliegt verschiedenen, sich ändernden Risiken. Zu den unerwünschten Stoffen zählen Rückstände wie Tierarzneimittel und Pflanzenschutzmittel sowie Kontaminanten wie (Halb‑)Metalle, persistente organische Kontaminanten, Prozesskontaminanten, aber auch natürliche Toxine. Menschliche Aktivitäten haben häufig einen direkten oder indirekten Einfluss auf den Eintrag und das Vorkommen dieser Stoffe in Lebens- und Futtermitteln. Hinzu kommt, dass einige dieser Stoffe die Eigenschaft besitzen, sich entlang des Pfades Boden/Wasser – Pflanze – (Nutz‑)Tier – Lebensmittel (tierischer Herkunft) anzureichern. Hier werden häufig die Begriffe „Transfer“ oder „Carry Over“ verwendet.

Gemäß Codex Alimentarius, WHO und FAO handelt es sich bei Carry Over um die Verschleppung der Kontamination eines Materials oder Produkts durch die Verwendung eines Geräts oder Ausstattungsgegenstandes in ein anderes Material oder Produkt. Transfer hingegen wird definiert als Übertragung einer chemischen oder biologischen Gefahr (einschließlich Biotransformationsprodukte) vom Futter eines der Lebensmittelgewinnung dienenden Tieres auf ein essbares Produkt des Tieres. Beispielsweise stellt der ungewollte Abrieb aus dem Futtermischer in ein Futtermittel einen Carry Over dar, der Übergang von Stoffen in ein tierisches Lebensmittel nach oraler Aufnahme einen Transfer [3–5].

Im Folgenden werden ausgewählte Beispiele von chemischen Kontaminanten und die Mechanismen von Eintrag und Weitergabe in Waren- und Nahrungsketten vorgestellt.

### (Halb‑)Metalle

Elemente wie Arsen, Blei, Cadmium und Quecksilber sind Beispiele für (Halb‑)Metalle, welche natürlich vorkommen, aber auch infolge menschlicher Aktivität, wie etwa durch Landwirtschaft und Industrie, ubiquitär in der Umwelt verbreitet sind [6–8]. Der Eintrag in die Nahrungskette erfolgt insbesondere durch die Aufnahme aus dem Boden, aus Wasser oder aus atmosphärischen Ablagerungen in pflanzliche und anschließend über die Nahrungskette auch in tierische Lebens- und Futtermittel. Das Vorkommen innerhalb der Nahrungskette hängt von vielen Faktoren ab, wie z. B. von den physikalisch-chemischen Eigenschaften des (Halb‑)Metalls und somit seiner Bioverfügbarkeit in Böden, Wasser, Pflanzen und Tieren (trophischer Transfer). Relevant sind aber auch die metabolische Stabilität der Verbindungen, die Verteilung und die Bioakkumulation in verschiedenen Spezies und in Geweben. Diese Faktoren können zu einer Exposition beitragen, die schließlich potenziell schädliche Auswirkungen auf die Gesundheit von Tieren und Menschen haben kann [6, 7].

Der Mensch kann die (Halb‑)Metalle über eine breite Palette von Lebensmitteln aufnehmen, darunter Produkte pflanzlichen (Getreide, Gemüse, essbare Wurzeln und Knollen, Pilze, Ölsaaten usw.) und tierischen Ursprungs (Fische, Krustentiere, Weichtiere, Fleisch und Innereien; [8–12]). Neuere Untersuchungen zum Vorkommen von (Halb‑)Metallen in Lebensmitteln im Rahmen der amtlichen Überwachung oder der BfR-MEAL-Studie des Bundesinstituts für Risikobewertung (MEAL: Mahlzeiten für die Expositionsschätzung und Analytik von Lebensmitteln) zeigen, dass in bestimmten Lebensmitteln höhere Konzentrationen zu erwarten sind [13–15].

Ein bekanntes Beispiel ist Cadmium in Kakao: Geogene, klimatische und eine Reihe weiterer Einflussfaktoren führen dazu, dass in den weltweiten Hauptanbauregionen für Kakao unterschiedliche Cadmiumgehalte in Kakaobohnen zu erwarten sind. Es wurden bereits diverse Maßnahmen (einschließlich der Festlegung von gesetzlichen Höchstgehalten) identifiziert, um die Einträge von Cadmium in Kakao zu minimieren. Von fast noch größerer Bedeutung ist diese Minimierung in Getreide, da aufgrund der Verzehrgewohnheiten selbst niedrige Cadmiumgehalte in Getreide beträchtlich zur Gesamtexposition beitragen können. Darüber hinaus hat Cadmium im menschlichen und tierischen Organismus eine extrem lange Halbwertzeit, so dass auch bestimmte Lebensmittel tierischer Herkunft (v. a. Innereien) teils sehr hohe Cadmiumgehalte aufweisen können.

Über die Verwendung bestimmter Düngemittel (z. B. Phosphate) kann Cadmium in den Boden und schließlich in die Pflanze und somit in Lebens- und Futtermittel gelangen. Die weltweiten Phosphatreserven sind nicht nur insgesamt begrenzt, sondern enthalten auch unterschiedlich hohe Konzentrationen an Cadmium. Lieferengpässe bei Rohphosphaten mit sehr geringen Cadmiumgehalten (z. B. wie aktuell aus Russland) können indirekt dazu führen, dass auf andere Phosphatquellen zurückgegriffen werden muss [16].

Die Gesundheit von Umwelt, Tier und Mensch sind dabei eng miteinander verknüpft. So kann das Vorkommen von (Halb‑)Metallen in der Umwelt sowie in der Nahrungskette so beispielsweise durch das Begrenzen industrieller Emissionen, Veränderungen der landwirtschaftlichen Praxis oder die Regulation von zulässigen Gehalten in Umweltmedien und Lebensmitteln minimiert werden. Dabei spielt die interdisziplinäre, aber auch internationale Zusammenarbeit eine zentrale Rolle, wie in der Codex-Alimentarius-Kommission (CAC) oder im Codex-Ausschuss für Kontaminanten in Lebensmitteln (Codex Committee on Contaminants in Food – CCCF).

### Persistente organische Kontaminanten

Zu den persistenten organischen Kontaminanten (engl. Persistent Organic Pollutants – POP) gehören sowohl synthetisch hergestellte Industriechemikalien als auch bei Verbrennungsprozessen unbeabsichtigt gebildete Verbindungen. Beispiele sind polychlorierte Biphenyle (PCB), polychlorierte Dibenzo-p-dioxine und -furane (PCDD/PCDF, kurz „Dioxine“), Per- und Polyfluoralkylsubstanzen (PFAS), Hexachlorbenzol (HCB), Organochlorinsektizide, polybromierte Diphenylether (PBDE) und polychlorierte Naphthaline. Diese Gruppe von Kontaminanten zeichnet sich durch eine hohe Langlebigkeit in der Umwelt, die Fähigkeit zur Bioakkumulation und ein Potenzial für schädliche Wirkungen auf Mensch und Tier aus. Aufgrund der Persistenz in der Umwelt in Verbindung mit einem weiträumigen Transport kann eine Exposition gegenüber diesen Stoffen auch dort erfolgen, wo sie ursprünglich nicht verwendet wurden, und zu einem Zeitpunkt, zu dem die bewusste Anwendung seit Langem reguliert oder beendet ist [17, 18]. Die Minimierung der Exposition erfordert im Sinne des One-Health-Ansatzes die interdisziplinäre Zusammenarbeit unter anderem im veterinär- und humanmedizinischen, toxikologischen sowie agrar- und umweltwissenschaftlichen Bereich.

#### Dioxine und dioxinähnliche polychlorierte Biphenyle (dl PCB)

Zu den wahrscheinlich am besten untersuchten und charakterisierten persistenten organischen Kontaminanten in Lebens- und Futtermitteln gehören die polychlorierten Dibenzo-p-dioxine und -furane (PCDD/PCDF, kurz „Dioxine“) und dioxinähnlichen polychlorierten Biphenyle (dl-PCB). Aufgrund ihrer weltweiten Verteilung sowie ihrer extremen Persistenz in der Umwelt können sie an verschiedensten Stellen der Nahrungskette nachgewiesen werden. Futtermittel sind dabei eine wichtige Eintragsquelle [19]. Eine Vielzahl von Kontaminationsgeschehen von Lebensmitteln tierischer Herkunft über kontaminierte Futtermittel ist in Europa in den letzten 30 Jahren dokumentiert worden [17]. Ergebnisse im Rahmen der amtlichen Überwachung oder der BfR-MEAL-Studie zeigen, dass Lebensmittel tierischer Herkunft auch ohne spezifische Kontaminationsgeschehen höhere Gehalte an Dioxinen und dl-PCB aufweisen können [20]. Durch entsprechende Maßnahmen sollen die gesundheitlichen Risiken durch diese Kontaminanten weiter reduziert werden. Global gehandelte Agrarrohstoffe (z. B. Futtermittel), weiterführende Untersuchungen zum Transfer aus Futtermitteln in Lebensmittel tierischer Herkunft sowie die Rückverfolgbarkeit von Kontaminationsgeschehen anhand charakteristischer „Muster“ von Dioxin-Kongeneren sind in Betracht zu ziehen, um Eintragsquellen zu identifizieren und zu unterbinden [21–23].

#### Per- und Polyfluoralkylsubstanzen (PFAS)

Nach ihrem Eintrag in die Umwelt können PFAS über sehr lange Zeiträume dort persistieren. Die Industriechemikalien verfügen über ein Potenzial zum weiträumigen Transport über Umweltmedien bis in entlegene Gebiete der Erde, in denen sie nicht eingesetzt werden, wie z. B. der Arktis. PFAS sind weltweit in Gewässern, Böden, Pflanzen, Tieren und auch im menschlichen Körper nachweisbar und können sich in der Nahrungskette Pflanze/Futtermittel/Nutztier/Mensch anreichern. Für die Exposition des Menschen spielt daher der orale Pfad eine große Rolle [18].

Die Gruppe der PFAS umfasst alle Verbindungen mit mindestens einer vollständig fluorierten Methyl- oder Methylengruppe. Die Organisation für wirtschaftliche Zusammenarbeit und Entwicklung (OECD) listet über 4300 CAS-Nummern^1^ als PFAS [24]. Die einzelnen Verbindungen unterscheiden sich sowohl in ihrer Mobilität in Umweltmedien als auch in ihrem Anreicherungsverhalten in der Nahrungskette. Dies führt – zusätzlich zu den verschiedenen Eintragsquellen in die Umwelt – zu heterogenen Mustern und Konzentrationen an PFAS. In Deutschland sind mehrere Regionen bekannt, in denen besondere Einträge in die Umwelt zu langfristig besonders hohen Gehalten an PFAS in Böden, Gewässern und zum Teil in dort regional erzeugten Lebensmitteln geführt haben.

So haben Bodenverunreinigungen durch Klärschlämme aus der Papierindustrie zu Einträgen von PFAS in Böden und Gewässer im Hochsauerlandkreis unter anderem zu erhöhten PFAS-Gehalten im Trinkwasser einiger Regionen bis zum Jahr 2006 geführt, die auch in erhöhten Gehalten im Blutserum bei Teilen der Bevölkerung resultierten [25, 26]. Ähnlich wurden PFAS im Kreis Rastatt durch die Aufbringung verunreinigter Kompostgemische auf landwirtschaftliche Nutzflächen in die Nahrungskette eingetragen [27].

Die Verwendung von PFAS-haltigen Löschschäumen führte zu Einträgen von PFAS in Böden, Grund- und Oberflächengewässer im Umfeld mehrerer Flughäfen wie dem Düsseldorfer Flughafen und dem Bundeswehrflughafen Manching. Dies gilt auch für die Kontamination der Luft im Umfeld einer ehemaligen Produktionsstätte von PFAS in Bayern (Gendorf; [28]). Anhand der Dioxin‑, PCB- und PFAS-Problematik zeigen sich eindrücklich die komplexen und vor allem langfristigen Wechselwirkungen zwischen Mensch, Umwelt und Tier und die Wichtigkeit, auch zukünftig im Sinne des Vorsorgeprinzips zu handeln.

### Natürliche Toxine

Pilze und Pflanzen können natürliche Toxine enthalten, von denen eine Gefahr für die Gesundheit von Nutztieren und den Menschen u. a. über den Transfer aus Futtermitteln ausgehen kann. Feld- und Lagerpilze sind seit Langem bekannt dafür, dass sie eine Reihe von Mykotoxinen mit unterschiedlichen gesundheitlichen Wirkungen für Menschen und Tiere produzieren können. Einige dieser Mykotoxine können auch über Futtermittel in Lebensmittel tierischer Herkunft gelangen (z. B. Milch). Darüber hinaus produzieren viele Pflanzengattungen Sekundärmetabolite (Pflanzentoxine), oft als Reaktion auf äußere Einflüsse wie Hitze, Wassermangel oder Fressfeinde. Rückgänge bei Ernten von traditionellen Futterpflanzen, die vermehrte Ausbreitung sogenannter invasiver (und toxischer) Pflanzenarten oder durch Krisenereignisse gestörte Handelswege können dazu führen, dass potenziell toxische Pflanzenmetabolite in die Nahrungskette gelangen (z. B. direkt oder durch konservierte und anderweitig verarbeitete Futtermittel; [29]).

#### Pyrrolizidinalkaloide (PA)

Bei einer Vielzahl von Pflanzengattungen sind Pyrrolizidinalkaloide (PA) als sekundäre Pflanzenstoffe zu finden. Besondere Aufmerksamkeit erfährt dabei das Jakobs-Kreuzkraut (*Jacobaea vulgaris*), dessen leberschädigende PA regelmäßig zur Vergiftung von weidenden Pferden und Rindern führen. Auch durch die Aufnahme von Heu oder Silagen kann es zu Vergiftungen kommen, wenn PA-haltige Pflanzen enthalten sind. Lebensmittel wie Salate, Tees [30] oder Gewürze [31] können PA-haltige Pflanzenteile enthalten und auch in Lebensmitteln tierischer Herkunft können durch den Übergang vom Futter in zum Beispiel die Milch PA-Gehalte nachgewiesen werden [32]. Vor diesem Hintergrund und der Tatsache, dass selbst geringe Aufnahmemengen mit einer Erhöhung des gesundheitlichen Risikos verbunden sein können, gilt nach wie vor die Empfehlung, die Aufnahme dieser Substanzen so weit zu minimieren, wie dies vernünftigerweise erreichbar ist. Im Sinne des One-Health-Ansatzes sollte ein besonderes Augenmerk auf potenzielle Eintragspfade in die Nahrungskette gelegt werden.

#### Tropanalkaloide

Der gemeine Stechapfel (*Datura stramonium*) ist eine global vorkommende Pflanze, welche zunehmend in landwirtschaftlich genutzte Flächen einwandert. Alle Teile des Stechapfels enthalten sogenannte Tropanalkaloide, von denen Atropin (ein racemisches Gemisch der Isomere (−)/(+)-Hyocyamin) und Scopolamin die mengenmäßig bedeutendsten darstellen [33]. Bis heute sind etliche Fälle dokumentiert, bei denen Kontaminationen von Lebens- und Futtermitteln mit (Teilen, Samen des) Stechapfel(s) negative gesundheitliche Auswirkungen beim Menschen hatten [29]. Insbesondere das Vorkommen von Tropanalkaloiden in global gehandelten Agrarrohstoffen wie Sojaschroten, Lein oder Getreide stellt eine besondere Herausforderung für die amtliche Kontrolle sowie die Risikobewertung dar. In Europa gelten für Datura-Samen im Rahmen der Richtlinie 2002/32 über unerwünschte Stoffe in der Tierernährung zwar Höchstgehalte, jedoch können auch hofeigene Futtermittel wie mit Stechapfel kontaminierte Maissilage zu gesundheitlichen Problemen bei Nutztieren führen [34]. Auch wenn es sich dabei um ein lokal begrenztes Geschehen handelte, sollten neben den direkten Auswirkungen auf die Tiergesundheit auch Aspekte der Warenketten tierischer Lebensmittel zukünftig in Betracht gezogen werden – kürzlich konnte gezeigt werden, dass ein Transfer von Tropanalkaloiden in die Milch von Kühen möglich ist [35].

#### Mykotoxine

Bei Mykotoxinen handelt es sich um sekundäre Stoffwechselprodukte, die von toxinbildenden Pilzspezies produziert werden, wobei *Fusarium* spp., *Penicillium* spp. und *Aspergillus* spp. in Europa die am häufigsten vorkommenden Spezies sind. Sie kommen überwiegend in Getreide, Gewürzen, Ölsaaten, Nüssen und Früchten vor und sind daher sowohl für die menschliche Gesundheit als auch für die von Nutztieren von Bedeutung. In Abhängigkeit von der jeweiligen Toxingruppe können überwiegend chronische, wie beispielsweise hämatopoetische, immunotoxische, reproduktionstoxische, leber- und nierenschädigende oder sogar kanzerogene Effekte, aber auch akute Effekte induziert werden [36].

Mykotoxine können sowohl direkt auf dem Feld als auch bei der Lagerung oder dem Transport entstehen. Entlang der Warenkette kommen Mykotoxine nicht ausschließlich in der freien Form vor, sondern – abhängig von den äußeren Bedingungen – in verschiedensten Modifikationen, welche unter dem Oberbegriff „modifizierte Mykotoxine“ zusammengefasst werden. Man unterscheidet dabei zwischen matrixassoziierten Mykotoxinen und solchen, die biologisch (z. B. in der Pilzspezies oder in der Pflanze) oder aber chemisch (z. B. während der thermischen Prozessierung) entstehen können. Im Hinblick auf den Verbraucherschutz und die Tiergesundheit ist zu beachten, dass die modifizierten Formen im Vergleich zur freien Form sowohl ein höheres als auch ein geringeres toxikologisches Potenzial aufweisen können und demnach u. U. ein zusätzliches Risiko für die Gesundheit darstellen können [37].

Neben pflanzlichen Lebens- und Futtermitteln können ferner auch Lebensmittel tierischen Ursprungs Mykotoxine enthalten. Ein Beispiel hierfür ist Ochratoxin A, das bei der Reifung und Lagerung von Schinken oder Käse entstehen kann [38, 39]. Daneben gibt es auch Hinweise darauf, dass ein Transfer von Deoxynivalenol oder Zearalenon über das Futtermittel in das erzeugte tierische Produkt erfolgen kann [40, 41]. Eine Ausnahme bildet hierbei die vielfach nachgewiesene enzymatische Umwandlung von z. B. in Futtermitteln vorhandenem Aflatoxin B1 zu Aflatoxin M1 in der Leber und der anschließende Übergang von Aflatoxin M1 in Milch, wie z. B. Kuhmilch [42, 43]. Neuere Untersuchungen zeigen, dass klimatische Veränderungen in Südeuropa dazu führen, dass mit vermehrtem Auftreten von Aflatoxin B1 gerechnet werden kann [44]. Die Kontamination von Futtermitteln mit Aflatoxin B1 stellt ein klassisches Beispiel dar, dass Klimawandel, globaler Handel von Agrarrohstoffen sowie die gesundheitlichen Risiken für Mensch und Tier ganzheitlich im Sinne des One-Health-Gedankens betrachtet werden sollten [45].

## Regionalität versus Globalität im Diskurs

Seit einigen Jahren wird einer regionalen Lebensmittelerzeugung und den damit einhergehenden kürzeren Lieferketten mehr Bedeutung beigemessen. Dies wirkt sich auch auf Angebot und Nachfrage im Bereich der Produktion von pflanzlichen und tierischen Lebensmitteln aus. In der Europäischen Union (EU) wurde daher vor 10 Jahren ein Rahmenprogramm verabschiedet, welches kurze Lieferwege bei der Lebensmittelproduktion („short food supply chains“ – SFSC) unterstützt: die Reform der Europäischen Agrarpolitik (Common Agricultural Policy – CAP; [46, 47]).

In Zeiten von Krisen gewinnen eine regionale Versorgung und ein hoher Selbstversorgungsgrad weiter an Bedeutung. Dies spiegelt sich auch in der Wahrnehmung von möglichen gesundheitlichen Risiken seitens der Bevölkerung wider. Eine Online-Umfrage des BfR, die im September 2022 mit Verbraucherinnen und Verbrauchern ohne Bezug zu Ernährung von Nutztieren (*n* = 1000) sowie Landwirtinnen und Landwirten (*n* = 250) aus Deutschland durchgeführt wurde, hat gezeigt, dass die Sicherheit von Futtermitteln, die in Deutschland genutzt werden, unterschiedlich nach ihrer Herkunft eingeschätzt wird. Futtermittel aus eigener Erzeugung der Landwirte sowie zugekaufte Futtermittel deutscher Herkunft werden von der Mehrheit der Befragten als (sehr) sicher eingestuft. Futtermittel mit Herkunft EU-Ausland hingegen werden von ca. einem Drittel der Befragten als (sehr) sicher und zugekaufte Futtermittel mit Herkunft außerhalb der EU von lediglich 13 % bzw. 14 % der Befragten als (sehr) sicher eingeschätzt (unveröffentlichte Daten). Dies zeigt, dass der Wahrnehmung von gesundheitlichen Risiken in Bezug auf Lebens- und Futtermittel zukünftig noch mehr Beachtung beigemessen werden sollte.

Trotz des in den Anfängen befindlichen Wandels der Warenketten von global zurück zu regional werden nach wie vor große Mengen an eiweißreichen Agrarrohstoffen (z. B. Ölsaaten und die daraus gewonnenen Schrote) in die EU importiert. Wesentliche Faktoren, wie beispielsweise die Nachfrage nach Lebensmitteln tierischer Herkunft, hohe Erträge beim Anbau anderer Agrarrohstoffe wie Getreide, das Verbot der Verfütterung von Tiermehlen, sowie internationale Handelsabkommen haben zu dieser Situation beigetragen [48]. Die Versorgung Europas mit einheimischen hochwertigen Proteinquellen wie Soja, Raps und Leguminosen hat daher in den letzten Jahren an Bedeutung gewonnen. Dennoch besteht weiterhin großes Potenzial, die Selbstversorgung mit hochwertigem Eiweiß in Europa zu optimieren. Derzeit ist die bedarfsgerechte Fütterung der Nutztierbestände in der EU und somit auch in Deutschland aktuell nur durch den weltweiten Bezug von Futtermitteln und Futtermittelzusatzstoffen möglich. Das folgende Beispiel soll dies noch einmal verdeutlichen: Für die optimierte bedarfsgerechte Versorgung von Nutztieren sind neben qualitativ hochwertigen und sicheren Einzelfuttermitteln insbesondere Futtermittelzusatzstoffe wichtig. Diese sind beispielsweise für den tierischen Organismus essenzielle Substanzen wie Aminosäuren, Vitamine, Enzyme oder Spurenelemente. Die Zugabe erfolgt vielfach tierartenspezifisch und orientiert sich am Bedarf. So spielt die Aminosäure Methionin insbesondere in der Legehennenfütterung eine wichtige Rolle, während Lysin beispielsweise in der bedarfsgerechten Fütterung von Schweinen benötigt wird.

Die SARS-CoV-2-Pandemie hat eindrucksvoll gezeigt, wie stark die Verfügbarkeit global gehandelter Stoffe vom internationalen Handel abhängig ist. Der Markt für Futtermittelzusatzstoffe, wie z. B. Lysin, war geprägt von Lieferengpässen und fehlenden Containern, die die Frachtraten in die Höhe trieben, von langen Lieferzeiten sowie Preisanstiegen. Das Beispiel zeigt eindrücklich, dass durch das Wegbrechen globaler Lieferketten die bedarfsgerechte Versorgung von Nutztieren in Deutschland unter Umständen nicht mehr gewährleistet werden kann. Welche Auswirkungen das auf die Tiergesundheit, den Tierschutz oder auch die Produktion qualitativ hochwertiger und sicherer Lebensmittel hat, sollte zukünftig eingehender betrachtet werden.

## Die Erzeugung pflanzlicher und tierischer Lebensmittel im Wandel

Klimawandel, Pandemie, Rohstoffknappheit, Energiemangellage – um nur einige aktuelle Krisen zu nennen – bewirken kurz-, mittel- und langfristig einen Wandel in unseren Futter- und Lebensmittelwarenketten. Was aufgrund jahrelanger Erfahrung als sicher galt, kann von einem Tag auf den anderen unsicher sein und eine Kaskade an Folgereaktionen nach sich ziehen. Systeme wie das europäische Schnellwarnsystem für Lebens- und Futtermittel (Rapid Alert System für Food and Feed – RASFF; [49]) sowie das Frühwarnsystem „Import Screening for the Anticipation of Feed Risks“ (ISAR) des Bayerischen Landesamts für Gesundheit und Lebensmittelsicherheit (LGL; [50]) können dabei helfen, kritische Warenketten zeitnah zu erfassen und entsprechende Maßnahmen zu etablieren.

Wie vielfältig Auswirkungen einer Krise sein können, hat die SARS-CoV-2-Pandemie gezeigt. Restriktionen wie Lockdowns, Reisebeschränkungen oder auch erhöhter Krankenstand bzw. Quarantänemaßnahmen können die gesamte Lebensmittelkette von Primärproduktion sowie die Weiterverarbeitung und Bereitstellung der Produkte für Verbraucherinnen und Verbraucher beeinträchtigen. Lieferketten können gravierend gestört oder ganz unterbrochen und das weitgehend ausgewogene Verhältnis zwischen Angebot und Nachfrage empfindlich verschoben werden. Das gilt sowohl für Produkte im Handel (z. B. Nudeln, Tomatenkonserven, Mehl, Hefe) als auch Vermarktungswege (z. B. Online-Handel). Veränderte und verzögerte Lieferketten können Gesundheitsgefahren sowie – aufgrund gestiegener Preise sowie Mangel – Anreize zu Verfälschungen darstellen. Die Warenkette tierische Lebensmittel wurde durch die Pandemie maßgeblich beeinflusst, da Fleischverarbeitungsbetriebe an verschiedenen Standorten weltweit, darunter auch in Deutschland, aufgrund von COVID-19-Ausbrüchen unter den Angestellten und auferlegten Reisebeschränkungen für saisonales Personal schließen mussten. Eine direkte Folge der Schließungen ist der Rückstau bei den Nutztiererzeugern, mit Konsequenzen wie unzureichendes Tierwohl durch Platzknappheit, nicht optimaler Futterverwertung und Ertragseinbußen.

Seit Februar 2022 wurde die deutsche Landwirtschaft durch die Situation in der Ukraine mit zahlreichen neuen Herausforderungen konfrontiert. So verringerte sich die Verfügbarkeit von Agrargütern – vorrangig eiweißreichen Futtermitteln, aber auch Getreide – aus der Krisenregion, es kam zu Einschränkungen auf dem Logistiksektor (z. B. nicht nutzbare Infrastruktur, Personalknappheit), zur Verknappung von Energie für Landwirtschaft, Lebens- und Futtermittelindustrie sowie zur Verteuerung und Verknappung von Düngemitteln. Die Verfügbarkeit von Nahrungs‑, Futter- und Düngemitteln wird in der EU aktuell als sichergestellt angesehen. Die EU ist weitgehend autark. Dennoch wirkt sich der Rückgang der Einfuhren von Mais, Weizen, Raps sowie Sonnenblumenöl und -schrot aus der Ukraine auf die Futter- und Lebensmittelpreise und auf die Lebensmittelindustrie der EU aus. Aufgrund der sich verändernden Verfügbarkeit und den Änderungen von Lieferketten können sich (neue) Risiken entlang der Warenkette ergeben. Mangelsituation und Preissteigerungen dürfen sich jedoch nicht negativ auf die Sicherheit von Futtermitteln sowie Lebensmitteln auswirken.

## Fazit

Die Lebensmittelsicherheit ist eine wichtige Säule des One-Health-Ansatzes und von überragender Bedeutung für die menschliche Gesundheit. Ebenso ist jedoch auch die Versorgung von Nutztieren mit qualitativ hochwertigen und sicheren Futtermitteln einerseits wichtig für die Tiergesundheit, andererseits aber auch von großer Bedeutung für die Sicherheit von Lebensmitteln tierischer Herkunft. Gesundheitsgefährdende Stoffe und Krankheitserreger können aus Futtermitteln in Lebensmittel übergehen, wobei unterschiedliche Faktoren wie Umweltbedingungen, Herkunft, Transport, Lagerung und Weiterverarbeitung einen Einfluss auf deren Vorkommen und somit die menschliche und tierische Gesundheit haben können. Klimawandel, Pandemien, Rohstoffknappheit oder Energiemangellagen sind nur einige Aspekte, die einen Einfluss auf die Verfügbarkeit, Zusammensetzung und letztlich die Qualität von Lebens- und Futtermitteln haben. Eine große Rolle spielen dabei auch die globalen Warenketten, die zunehmend im Hinblick auf die Umweltauswirkungen als auch die Resilienz in Krisengeschehen diskutiert werden. Diese Aspekte zukünftig im Rahmen des One-Health-Ansatzes zu beleuchten, zu verstehen und zu kommunizieren, ist eine der großen Aufgaben aller Akteure im Verlauf der Lebensmittelkette.

## Footnotes

1 CAS (Chemical Abstracts Service) ist ein internationaler Bezeichnungsstandard für chemische Stoffe.
